# Prevalence and Predictors of the Burnout Syndrome in Medical Students of Karachi, Pakistan

**DOI:** 10.7759/cureus.4879

**Published:** 2019-06-11

**Authors:** Arifa A Asghar, Arisha Faiq, Shiza Shafique, Faiza Siddiqui, Noureen Asghar, Shanza Malik, Syeda Duaa Kamal, Ayesha Hanif, Muhammad F Qasmani, Syed U Ali, Summaiya Munim, Alishba Solangi, Amna Zafar, Muhammad O Sohail, Abeeha Aimen

**Affiliations:** 1 Internal Medicine, Dow University of Health Sciences (DUHS), Karachi, PAK; 2 Internal Medicine, Bahria University Medical and Dental College, Karachi, PAK

**Keywords:** burnout syndrome, medical students, psychological effect, mbi-hss, pakistan, suicide, suicide rate

## Abstract

Objectives

Burnout is a psychophysiological syndrome, consisting of a triad of emotional and physical exhaustion, exhibition of impersonal attitude and loss of a sense of achievement for oneself. This study aimed to pinpoint its risk factors, measure its current prevalence in medical students of Karachi, Pakistan and accentuate the areas of focus to benefit the primary care-oriented community as a whole.

Methods

This cross-sectional study included responses from 600 medical students in Karachi (third to final year). A self-administered questionnaire using the Maslach Burnout Inventory-Human Services Survey (MBI-HSS), multi-dimensional mood state questionnaire and perceived stress scale was used, along with a section about burnout prevention assessment. Data were analyzed using SPSS version 24.0 (IBM Corp., Armonk, NY) and chi-square tests used to find significant associations.

Results

One-fifth (n=109, 18.2%) of our subjects were burned out. The syndrome was significantly observed in those who operated on insufficient sleep (p-value 0.028) and in those having anger management issues and non-dominating temperaments (p-value 0.05). Furthermore, it was statistically significant in those who gave up easily, in those who had no hobbies and had no time to exercise and pray (p-value <0.05). It was more prevalent in pupils of private medical colleges whereas two of its three constitutive factors, Emotional Exhaustion (p-value 0.03) and Personal Achievement (p-value <0.001) were significantly higher in pupils of public sector universities.

Conclusion

The deleterious repercussions of burnout syndrome warrant the need for extensive efforts towards the propagation of its awareness.

## Introduction

The rapid progress in the continuously transforming field of medicine, its associated unrelenting educational demands, long working hours and extreme low tolerance for mistakes, all pose a huge negative impact on medical students - predisposing them to psychological morbidity. With a reportedly weaker ‘coping reserve’ due to lesser time for self-care, these students frequently fall prey to some deleterious consequences, such as substance abuse and even suicide, with studies showing figures as high as one in every five burned out students taking their lives [1-2].

The term ‘burn out’ was initially introduced by an American psychologist, Herbert Freudenberger, in his research paper in 1974 where he described it as, ‘exhaustion due to the inability to cope with increasing work demands, manifested with headache, sleep disturbances, behavioral changes, and reduced cognition.’ Burnout is a psychophysiological syndrome, consisting of a triad of emotional and physical exhaustion, exhibition of impersonal attitude and loss of a sense of achievement for oneself [3]. It is reportedly more evident in healthcare professionals, as compared to employees of other fields [4].

Maslach and Jackson devised the Maslach Burnout Inventory (MBI) in 1981 to evaluate burnout, which remains to be the most frequently used tool to study it even today. Studies continue to show a varying range of percentage prevalence’s for the syndrome. A survey conducted in Atlanta, USA in 2010 displayed a prevalence of burnout ranging from 21% to 43% in medical students [5], while a similar one conducted in São Paulo showed that burnout was detected in 14.9% of the students and 57.7% showed a risk of developing the syndrome [6].

Being highly popular among most of the career professionals, burnout remains an interesting topic for researchers to study. Its enormous adverse impact on productivity, physical and mental health and its life harming sequelae, all raise the dire need of further exploration into the theme. In addressing the same, this study aimed to pinpoint the risk factors, measure the current prevalence of burnout in medical students of Karachi, Pakistan and accentuate the areas of focus to benefit the primary care-oriented community as a whole.

## Materials and methods

Sample and setting

This cross-sectional study was conducted among medical students of Karachi, Pakistan after approval from the concerned Institutional Review Board (IRB). Only medical students in the clinical years of school were recruited for the study, keeping students below the third year in the exclusion criteria. Socioeconomic status of participants ranged from medium to high. The sampling technique used was convenience sampling. The sample size required was of 384 subjects which was calculated using OpenEpi version 3.01 (www.openepi.com), to fulfill the objectives of our study at 95% confidence level. The sample size was calculated assuming a 50% prevalence of burnout syndrome and 5% bound of error. The sample was then inflated to 600 to account for non-respondents and incomplete questionnaires. The data were collected during the period of April to May, 2018. The entire procedure was carried out under the guidelines of the Helsinki declaration.

Data collection

Data were collected by a group of 15 researchers via hard copy and online questionnaires. To assess the validity of the questionnaire, a pilot study was conducted among 31 students. A total of 656 students were approached, out of which only 600 filled the questionnaire properly marking the cooperation rate at 91.5%. The interviewers chosen were fluent in English and Urdu to avoid any miscommunication with students - also, a proper dress code with white coat was strictly observed by all. Verbal, informed consent was obtained by participants before giving them the questionnaire. Some students refused to fill the form when we approached them in late university hours due to weariness. Students were assured by the researchers that their information would remain confidential. Incomplete forms were disqualified and no imputation method was used.

Questionnaire

A self-administered questionnaire in the English language was used. A researcher was always present to answer the queries of students while filling the questionnaires, if they had any. The questionnaire had three sections: the first section had demographic questions, the second consisted of questions to assess the burnout of the student using Maslach Burnout Inventory-Human Services Survey (MBI-HSS), multi-dimensional mood state questionnaire and perceived stress scale and the third section was about burnout prevention assessment.

Prevalence of burnout among students was assessed mainly through the MBI-HSS which consisted of 22 items in three subscales i.e., Emotional Exhaustion subscale, Depersonalization subscale, and Personal Achievement subscale. The responses to 22 questions were constructed on a 7-point, Likert scale - ranging from 0 (never) to 6 (always). MBI-HSS is known to have good reliability and validity. Classification into low, moderate and high scores is usually based on established cut off scores, but since there was no established burnout cut-off score available for Pakistan, each distribution of scale scores was divided into quartiles - high score was assigned the 75th percentile or higher, low score was in the 25th percentile or lower and the moderate score lay in between. Considering ‘Emotional Exhaustion + 1’ criterion, a high score on both Emotional Exhaustion subscale and the Depersonalization subscale; or a high score on the Emotional Exhaustion subscale and a low score on the Personal Achievement subscale was used to distinguish and categorize burnt out individuals from non-burnt out ones. In the third section, to assess various ways of prevention, 13 questions with options ‘yes,’ ‘no’ and ‘sometimes’ were added to the questionnaire.

Data analysis

Data were entered and analyzed using SPSS version 24.0 (IBM Corp., Armonk, NY). Frequency distribution was calculated for demographic data, such as gender and qualification. The association of burnout syndrome with different demographic and other social factors was assessed with the use of chi-square test and a p-value of 0.05 or less was considered as significant with confidence limit of 5%.

## Results

A total of 600 respondents with a mean age of 21.8 ± 1.1 years (range: 19-27) participated in this survey, majority (n=397, 66.2%) of them being females. The participants hailed from third, fourth and fifth years of four public (n=394, 65.7%) and nine private sector (n=206, 34.3%) medical universities; a staggering majority contributed by students of the tfourth year (n=318, 53%) and Dow Medical College (n=374, 62.3%). A minor number (n=10, 1.7%) of the respondents were involved in other paid professions (Table 1).

**Table 1:** Gender distribution of students on the basis of year of education, type of university, and occupation

Variable	Females (%)	Males (%)
Year of education		
Third	131 (21.8%)	62 (10.3%)
Fourth	209 (34.8%)	109 (18.2%)
Fifth	57 (9.6%)	32 (5.3%)
University		
1) Private		
- Aga Khan University	10 (1.7%)	18(3%)
- Bahria University Medical and Dental College	50 (8.3%)	20 (3.3%)
- Dow International Medical College	18 (3%)	9 (1.5%)
- Hamdard College of Medicine and Dentistry	-	1 (0.2%)
- Jinnah Medical and Dental College	12 (2%)	6 (1%)
- Liaquat National Medical College	13 (2.2%)	15 (2.5%)
2) Public		
- Dow Medical College	264 (44%)	110 (18.3%)
- Jinnah Sindh Medical University	5 (0.8%)	3 (0.5%)
- Karachi Medical and Dental College	9 (1.5%)	2 (0.3%)
- Shaheed Mohtarma Benazir Bhutto Medical University	1 (0.2%)	-
Occupation		
Businessman	-	1 (0.2%)
Freelancer	1 (0.2%)	-
Tutor/Teacher	3 (0.5%)	5 (0.8%)

According to the aforementioned “Emotional Exhaustion + 1” criterion, one-fifth (n=109, 18.2%) of the subjects were found to be burned out. The mean Emotional Exhaustion score was 17.3 ± 9.2, that of Depersonalization was 11.6 ± 7.5 while Personal Achievement had a mean of 22.5 ± 9.2, their ranges being the same i.e. 0-42. According to the 25th and 75th percentile of their respective scores, all three dimensions were assorted into low, moderate and high sub-groups; over two-fifth of the participants were found to be corresponding to the moderate category in all three dimensions (Table 2).

**Table 2:** Frequency and percentage of Maslach Burnout Inventory (MBI) dimensions

MBI Dimensions (scores)	Females (%)	Males (%)
Emotional Exhaustion		
Low (0-10)	98 (16.3%)	64 (10.7%)
Moderate (11-12)	175 (29.2%)	101 (16.8%)
High (23-42)	124 (20.7%)	38 (6.3%)
Depersonalization		
Low (0-6)	120 (20%)	50 (8.4%)
Moderate (7-15)	170 (28.3%)	89 (14.8%)
High (16-42)	107 (17.8%)	64 (10.7%)
Personal Achievement		
Low (0-15)	119 (19.8%)	45 (7.5%)
Moderate (16-29)	179 (29.8%)	102 (17.1%)
High (30-42)	99 (16.5%)	56 (9.3%)

Burnout syndrome was significantly observed in those who slept for less than six hours (p-value = 0.028) and did not share their concerns and problems with anyone (p-value= 0.037). It also showed a statistical significance (p-value <0.05) in subjects incapable of controlling their anger, those having no hobbies due to time constraints or the mental energy for recreational pursuits. People who were unable to develop their abilities due to lack of opportunities, respondents with a non-dominating temperament and individuals unable to spend a full day to their liking or embark on a vacation once a year, all bore a statistically compelling association with the burnout syndrome (p-value <0.05). Furthermore, the syndrome significantly coexisted (p-value <0.05) in those who did not exercise, pray, indulge in humor, gave up when things spiraled out of their control and had no aspirations regarding their future (Table 3). However, when paired with gender, year of medical education and public/private sector universities, burnout syndrome yielded an insignificant association (p-values = 0.188, 0.534 and 0.566 respectively).

**Table 3:** Burnout syndrome prevention assessment

	Burned out	Not burned out	P-values
Do you have a full day off to do what you like?			
Yes	21	152	
Sometimes	36	194	0.001
No	52	145	
Do you have time out for yourself to pray?			
Yes	54	381	
Sometimes	40	77	<0.001
No	15	33	
Do you have a good vacation about 3-4 weeks per year?			
Yes	41	218	
Sometimes	13	90	0.03
No	55	183	
Do you do some aerobic exercises?			
Yes	17	132	
Sometimes	29	110	0.047
No	63	249	
How well do you communicate?			
Excellent	18	118	
Fair	67	288	
Difficult	17	53	0.363
Poor	7	31	
Missing	-	1	
Do you have a dominating behavior?			
Yes	28	131	
Sometimes	33	198	0.037
No	49	161	
Are you diet conscious?			
Yes	26	138	
Sometimes	33	117	0.339
No	50	236	
Do you have control over your anger?			
Yes	32	230	
Sometimes	50	170	0.005
No	28	90	
Are you fond of humor? Does it work as a stress reliever?			
Yes	64	369	
Sometimes	35	90	0.002
No	10	32	
Do you have a creative hobby time?			
Yes	33	249	
Sometimes	36	122	<0.001
No	40	120	
Are you ambitious about your future?			
Yes	60	395	
Sometimes	36	72	<0.001
No	13	24	
Do you have extra space in mind to think about things other than work?			
Yes	66	362	
Sometimes	28	90	0.01
No	18	36	
Does your work provide you opportunities to develop your abilities?			
Yes	27	245	
Sometimes	41	156	<0.001
No	42	89	
When things do not work as they should, do you stop trying?			
Yes	21	48	
Sometimes	46	177	0.001
No	41	267	

Moreover, Emotional Exhaustion was significantly observed in females (p-value 0.005) and respondents pertaining to public sector universities (p-value = 0.03). Slumber of less than six hours contributed significantly to both Emotional Exhaustion (p-value <0.001) and Depersonalization (p-value = 0.008) whereas low and high scores in Personal Achievement were significantly discerned in those affiliated with public (p-value <0.001) and private (p-value <0.001) universities, respectively.

## Discussion

To the best of our knowledge, this is the first study that assesses the prevalence of burnout syndrome amongst medical students of Karachi, Pakistan. Our results showed a prevalence of 18.2% which indicates that out of every five participants in our study, one was burned-out.

Although this percentage is meaningful, it is lower than that reported in previous studies. Studies conducted among the medical students of Lahore, Pakistan, and Sweden, both reported a 47% prevalence of burnout in their sample populations [7-8], while Dyrbye et al. (2010) [9] reported a prevalence of 52.8% among American medical students. However, studies with more conservative criteria displayed lower prevalence’s, similar to our results. A survey conducted among medical students of Brazil reported a prevalence of 10.3% [10] and that amongst pediatrics clerkship students of Universidade Federal da Bahia-Brazil concluded a burnout prevalence of 14.5% [11]. This large variability in results reflects the difference in criteria used to define burnout syndrome and the variation in tools and applied cut off points. Our method of using all three dimensions that are part of MBI scale and applying ‘EE+1 criterion’ to obtain the results provided a fairly small chance (6.8%) of an incorrect qualification of burnout [12]. In addition, significant dissimilarity in sociocultural contexts and academic curriculum among medical schools may also have contributed to the above-mentioned variation in results. 

Furthermore, a separate analysis of each subscale revealed over two-fifths of participants as belonging to the moderate category, in each of the three dimensions. In relation to this, another plausible explanation for low burn out prevalence in our study is the presence of personal achievement in the moderate category which can counterbalance the negatives of Emotional Exhaustion and Depersonalization, offering a protective role. This might stem from the current system of clinical teachings in Pakistan which allows medical students (third year onwards) to rotate in a different clinical department every few weeks. During their time in each department, students are given minimum responsibilities of patient workload while maximum attention is given to their clinical learning. This consequently alleviates the perception of incompetence and emotional depletion which are linked to having a more involved role in patient workload [13] and responsibility.

Regarding demographic variables in our study, gender did not share a significant association with burnout syndrome. This finding, although similar to those in a few studies [5,14], was contrary to numerous others [7-8] that found significant associations of female sex with high burnout levels and exhaustion. One possible explanation for our negative result may be that the recent emergence of a female majority trend in medical schools has alleviated some of the burden previously faced by female students to equal or even outperform in a male-dominated learning environment. Additionally, males are under added pressure regarding parental expectations, being the main ‘breadwinners’ within Asian cultures even today. This, along with how females are known to have better social support systems, more rational decision-making abilities concerning priorities in life and for being more vocal about their psychosocial issues, all explain why a great number of males may be burned out, preventing a female majority [15]. However, Emotional Exhaustion was significantly observed in the female participants of our study. Previous studies have reported marked interpersonal sensitivity and self-rated depression in females [16], displayed as more females internalizing high stress after patient interactions and autopsy procedures, as compared to males [17]. These traits, coupled with juggling multiple social roles outside of medical education can deplete female students of energy and time, contributing to a sense of Emotional Exhaustion.

Moreover, upon comparison burnout syndrome was found to be more prevalent among medical students belonging to private sector universities. These students experience added pressures of more expensive tuition fees and stricter policies. Also, students enrolled in such universities tend to belong to relatively higher socioeconomic backgrounds and more educated families, reportedly striving to emulate perfectionistic attitudes and under even higher familial expectations [7]. In contrast, Emotional Exhaustion was significantly observed in medical students of public sector universities. The traditional medical education model of public sector universities includes outdated teaching strategies, lack of reliable mentorship [2], delayed patient contact and excessive emphasis on assessments, coupled with overwhelming academic load and limited resources. Eventually, this non-supportive climate drains students of their emotional resources. In addition, our results showed no association between year of education and burnout syndrome. A study amongst Swedish medical students [8] showed similar results, whereas numerous others [5,7,14] have shown pathological values that increased as students progressed in their clinical years - possibly due to the increasing impact of the uncertainty of career progression post-graduation. One plausible explanation for the lack of association in our study can be the low representation of fifth-year medical students (14.8%).

Among psychosocial factors related to burnout syndrome, our study found loneliness and sleep deprivation (that is less than six hours of sleep) to be predisposing stressors - in line with pre-existing literature [7,18-19]. Sleeping difficulties are linked to high failure rates, poor academic performance [20] and metabolic dysregulation [21], all of which can eventually become triggers for the development of adverse mental health and burnout syndrome. The syndrome significantly coexisted in those who did not exercise, the vice versa being stated by Dyrbye et al. (2017) [22] in their study. Additionally, a study conducted among male participants with high burnout scores found a significant reduction in burnout symptoms and perceived stress after a 12-week aerobic exercise program [23]. The syndrome also shared a significant association with those who do not pray. Physical and psychological health has been significantly associated with religiosity [24] which may help protect against adverse effects of stress in many ways like the development of a sense of coherence and promoting salutary health-related practices [25]. Our study did not show a significant association of the syndrome with diet-related health behaviors. This was in contrast to studies from Western countries, where more rampant alcohol consumption has been identified as an essential contributor to the burnout syndrome, whether indulged in for pleasure or as a coping strategy [26].

In addition to that, among personal attributes, inability to control anger was significantly related to burnout syndrome. Unlike anxiety and depression, anger has not been studied as extensively, when considering its association with burnout. Moreover, our results also showed that burnout syndrome shared a significant association with those who did not indulge in humor, embark on vacation or have a hobby. These coping strategies enable students to deal with perceptions of incompetence and disengagement by clearing their mental palate, boosting their self-esteem and encouraging them to socialize with others. Interestingly, we also found a significant association of the syndrome with non-dominating temperaments. This contrasted with previous studies that have portrayed a higher prevalence in those with ‘Type-A’ behavior, due to their increased impulsivity and competitiveness [7]. Burn out was also found to be significantly associated with characteristics like giving up easily in the face of challenges and a lack of aspirations regarding the future. Along with making it easier to understand the links between the syndrome, psychiatric disorders and suicidal ideations [1,9], this finding strengthened the points raised by a Lebanese study that explained how the delivery of suboptimal care, increased medical errors and a heightened desire to give up practice amongst these individuals makes burn out a human resources issue and a burden for the healthcare system as well [27].

Fortunately, however, Dyrbyre et al. (2008) [1] have found burn out to be a reversible condition which makes it essential to mention some recommendations to mitigate the predicament caused by the syndrome. To address this condition effectively, students and educators must first be mindful of it and have a sufficient understanding of its symptoms. Implementing educational programs in mindfulness and awareness has been said to potentially improve empathy and decrease burnout among primary care physicians [28]. In the same vein, initiatives to promote individual strategies to enhance students’ well-being are also recommended. These include counseling, social support, cognitive behavioral therapy, physical exercise, and relaxation techniques. Lastly, medical schools may use their esteemed position in society responsibly, to promote professional development and resilience among students by adopting strategies that can optimize the learning environment and thus, take the lead in bringing about this crucial change. Student wellness, mentoring and awareness programs should be launched, especially targeting coping mechanisms to prevent the formation of dysfunctional strategies (like substance abuse and alcohol consumption) and guiding students towards healthier solutions.

Limitations

Despite our best efforts, this study has some limitations. The cross-sectional design of this study is useful at the population level, but it limits the deduction of causality which highlights the need for longitudinal studies in the future. Important variables relevant to our region like academic curriculum, exam frequency, drug abuse, peer pressure, parental expectation [19] and financial burdens [7] were not included in this study. The difference in instruments used to measure burnout limits the comparability of our results with previous studies. Baseline values were not collected before entry into medical university and the sampling technique used was convenience sampling, thus resulting in unequal representation in terms of gender, number of students from each year of study and the respective categories of public and private sector universities. Students from the pre-clinical years were not included, thus the effect of the transition into clinical practice on burnout syndrome could not be assessed.

## Conclusions

Thus, the findings of our study clearly exhibit that the stress that plagues medical students and its psychological impact should be of major concern, with focused efforts being directed towards propagating awareness and promoting better standards of self-care and wellness among students. It is also emphasized that efforts be directed towards conducting prospective longitudinal studies to assess the effectiveness of preventive and proactive coping strategies through the course of medical education. This will enable accurate implementation of strategies to “vaccinate” students of this syndrome, early on in their education.
